# GP workforce crisis: what can we do now?

**DOI:** 10.3399/bjgp22X719225

**Published:** 2022-04-29

**Authors:** Laura Jefferson, Mike Holmes

**Affiliations:** Department of Health Sciences, University of York, York.; Haxby Group, York; Chair, Nimbuscare, York; Chair, Trustee Board, Royal College of General Practitioners, London; Honorary Professor, Department of Health Sciences, University of York, York.

## INTRODUCTION

Recent weeks have seen GPs giving evidence to a parliamentary committee on the crisis facing UK general practice, with particular focus being placed on the need to recruit and retain the workforce. GPs were dissatisfied and struggling before the COVID-19 pandemic,^1^^–^^3^ but, as a systematic review included in this issue suggests,^4^ this period has exposed GPs to additional pressures without the resources to manage them. Several commentaries have been made as to the sources of these pressures and there has been a call to action for policymakers to address growing issues of general practice capacity. UK GP workforce vacancy rates reveal one in seven GP posts are currently vacant,^5^ but while increasing the pool of doctors will take some time to achieve, here we consider potential solutions to support general practice in the immediate term and retain those doctors we have. In the spirit of CS Lewis: *‘You can’t go back and change the beginning, but you can start where you are and change the ending’*.

We use Michael West’s framework — the ABC of needs^6^^,^^7^ — to frame policy solutions for general practice using the concepts of autonomy, belonging, and contribution that have previously been shown to be integral to promoting the wellbeing of the health workforce. Our own research evidence^4^^,^^8^^,^^9^ highlights how these key characteristics were eroded during the COVID-19 pandemic, but understanding the importance of these facets may also offer future solutions.

## AUTONOMY AND CONTROL

GPs’ autonomy and ability to exert control over their working lives has been reduced during the COVID-19 pandemic as they adapted rapidly to evolving guidelines and responded to vaccine delivery programmes. GPs need to work in an environment that enables them to work effectively; in highly-functioning teams managing an achievable workload alongside the support of effective IT systems and in buildings that are fit for purpose. The pandemic has seen an increase in demand for care, with workload spiralling and emphasis on quicker and longer access. While this does require focus, we must not lose sight of the need for relational continuity. Given the pressure the service is under, perhaps practices need to consider how to prioritise continuity for those who would benefit from it most?

The use of technology has evolved the consultation; GPs must now be empowered to tailor its use so as to best meet the needs of their patients locally — it is unlikely that one size will fit all. Multiprofessional teams may offer some opportunities, but again, the solutions will not be universally applicable. The Additional Roles Reimbursement Scheme may offer support; however, challenges of demand and supply in coastal, rural, and deprived urban areas may widen health inequalities, as we have seen with GP recruitment.

## BELONGING

Doctors need to feel valued, respected, and supported in their work;^6^^,^^7^ yet experiences during the pandemic highlight real issues of fear and blame in general practice.^8^^,^^9^ GPs’ sense of belonging has been threatened further by remote working and increased pressures on teams.^8^ Supportive, compassionate leadership is needed at all levels. From the national to the regional and local practice levels, there are steps that could be taken to increase this sense of belonging.

The political and media-driven narrative that described the role of UK general practice during the pandemic did much to demoralise the profession and threatened their all so important relationship with patients.

Regionally, as we move towards the full implementation of Integrated Care Systems, the opportunity to work collaboratively across sectors with a real focus on patients and population health feels encouraging. We have seen some excellent examples of this during the pandemic; however, there is some nervousness within general practice about this transition, and greater understanding is needed about how this can enable general practice to really belong and contribute in an equitable way across the health system.

At practice level, poor morale and wellbeing is affecting all staff groups, not just clinicians. Progress has been made during the pandemic to develop a culture that promotes and nurtures staff wellbeing; however, there remains issues of presenteeism and a perceived tokenism to these measures. All staff in general practice need to feel it is ok to not be ok. Support is being offered from multiple avenues and schemes such as ‘Looking after you too’^10^ have been met with demonstrable success. A more overt shift towards a culture that allows time for and normalises the proactive pursuit of wellbeing is perhaps necessary if the profession’s sense of belonging is not to be eroded further.

## CONTRIBUTION

Doctors need to experience effectiveness and feel they are delivering valued outcomes.^6^^,^^7^ If doctors are to be attracted into general practice, the professions’ contribution must be understood and valued. We have heard successive Prime Ministers refer to general practice as the cornerstone of the NHS, and yet GPs have faced extreme public scrutiny and continue to feel undervalued.

The pandemic may offer an opportunity to build on the civic partnerships seen during the COVID-19 vaccination rollout; strengthening communities and public perceptions of primary care. The importance of patient participation and involvement in the development of Integrated Care Systems has been highlighted as an opportunity to reset relationships between the public and health services.^11^^,^^12^ Furthermore, integration across all sectors of health and social care has an important role to play in not only delivering effective outcomes, but also in creating a wider sense of mutual understanding of what each specialty does. Opportunities to work together in co-designed services could have real benefits and build on the previous calls to have general practice rotations as a standard for all post-graduate doctors.^13^

GPs are increasingly moving to work on a sessional basis, away from a commitment to a registered list of patients.^14^ Action is required in this space. New to general practice fellowship training schemes have been introduced to support recently-qualified GPs; in some areas these are being enhanced. In North Yorkshire, workload is protected allowing space to grow into the profession, develop interests, continue learning, and prevent burnout.^15^ These doctors feel valued and develop a connection with their profession and their patients.

At the other end of the career spectrum, we have seen an enormous contribution from retired doctors and nurses returning to the workforce during COVID-19. Many wish to continue and would like to see this programme evolve; perhaps widening to other roles. It would seem, anecdotally at least, that any progress here would be welcomed on the frontline.

## CONCLUSION

We have highlighted some of the workforce issues and opportunities for change in general practice, using the lens of clinicians’ need for autonomy, belonging, and contribution in their working lives. This is clearly a multifaceted, complex area and the solutions are not straightforward. Understanding and valuing the profession while listening to and addressing its needs might prevent a complete restructure and enable a more effective path towards better patient experience and outcomes.

There is an overarching need to invest in the workforce to deliver greater numbers of doctors and allied health professionals in general practice. An independent workforce planning strategy is needed, combined with improved data collection across general practice so research evidence can highlight the impact of these difficulties on service delivery and, ultimately, patient care.
